# Supplementary material for: Using the Edmonton Symptom Assessment System (ESAS) to Describe Symptom Burden Associated with Breast Cancer and Related Treatments: A Cross-Sectional Study
Source: Curr Oncol. 2025 Oct 24;32(11):598. doi: 10.3390/curroncol32110598 (PMC12651290; doi:10.3390/curroncol32110598)
Supplement: Supplementary file 1 [file curroncol-32-00598-s001.zip › curroncol-3831126-supplementary.pdf]

Table S1: The STROBE reporting checklist

For checking that observational epidemiology research articles can be understood and used by everyone

	Item Description	Location (or reason for not reporting)
**Title and abstract**		
1a. Indicate the study's design	Indicate the study's design with a commonly used term in the title or the abstract.	Title and Abstract (Methods section)
1b. Abstract	Provide in the abstract an informative and balanced summary of what was done and what was found.	Abstract
**Introduction**		
2. Background / rationale	Explain the scientific background and rationale for the investigation being reported.	Introduction, full section
3. Objectives	State specific objectives, including any prespecified hypotheses.	Introduction, last paragraph
**Methods**		
4. Study design	Present key elements of study design early in the paper.	Material and Methods, section 2.1. Research design and procedures
5. Setting	Describe the setting, locations, and relevant dates, including periods of recruitment, exposure, follow-up, and data collection.	Material and Methods, sections 2.1. Research design and procedures and 2.2. Setting and participants
6a. Eligibility criteria	**Cross-sectional study:** Give the eligibility criteria, and the sources and methods of selection of participants.	Material and Methods, section 2.2. Setting and participants
6b. Matching criteria	**Cohort study:** For matched studies, give matching criteria and number of exposed and unexposed. **Case-control study:** For matched studies, give matching criteria and the number of controls per case.	Not applicable
7. Variables	Clearly define all outcomes, exposures, predictors, potential confounders, and effect modifiers. Give diagnostic criteria, if applicable.	Material and Methods, sections 2.3. Symptom and functional status assessment, and 2.5. Statistical analysis
8. Data sources / measurement	For each variable of interest give sources of data and details of methods of assessment (measurement). Describe comparability of assessment methods if there is more than one group.	Material and Methods, sections 2.2. Setting and Participants, and 2.3.Symptom and Functional Status Assessment.

9. Bias	Describe any efforts to address potential sources of bias.	Material and Methods, sections 2.1. Research Design and Procedures and 2.2. Setting and Participants.
10. Study size	Explain how the study size was arrived at.	Material and Methods, section 2.4. Sample size.
11. Quantitative variables	Explain how quantitative variables were handled in the analyses. If applicable, describe which groupings were chosen, and why.	Material and Methods, Section 2.4. Statistical analysis.
12a. Statistical methods	Describe all statistical methods, including those used to control for confounding.	Material and Methods, Section 2.4. Statistical analysis.
12b. Statistical methods – subgroups and interactions	Describe any methods used to examine subgroups and interactions.	Material and Methods, Section 2.4. Statistical analysis.
12c. Statistical methods – missing data	Explain how missing data were addressed.	Missing data were minimal for most variables. The number of patients with missing responses was reported in the relevant tables (see Table 1). No imputation was performed.
12d. Statistical methods – sampling strategy	**Cross-sectional study:** If applicable, describe analytical methods taking account of sampling strategy.	Because this study used a convenience sample of consecutively recruited patients rather than a probability-based design, no statistical weighting or adjustment for sampling design was required. All available participants who met eligibility criteria during the study period were included in the analyses.
12e. Statistical methods – sensitivity analyses	Describe any sensitivity analyses.	No formal sensitivity analyses were conducted. This study was primarily descriptive and exploratory in nature, based on a fixed convenience sample.
**Results**		
13a. Participant numbers	Report the numbers of individuals at each stage of the study—e.g., numbers potentially eligible, examined for eligibility, confirmed eligible, included in the study, completing follow-up, and analysed; Consider use of a flow diagram.	Results section, first paragraph and Table 1.

13b. Participants – non-participation	Give reasons for non-participation at each stage.	Reasons for non-participation were not systematically collected, as this was a secondary analysis of an existing dataset derived from routine clinical symptom screening. However, we compared demographic and clinical characteristics between respondents and non-respondents (Table 1) to assess potential differences.
13c. Participants – flow diagram	Consider use of a flow diagram.	A flow diagram was not included because the study design involved a single assessment point with no follow-up stages or exclusions beyond questionnaire completion, which limited the added value of a flow diagram.
14a. Descriptive data – participant characteristics	Give characteristics of study participants (e.g., demographic, clinical, social) and information on exposures and potential confounders. Present the information in a table.	Table 1
14b. Descriptive data – missing data	Indicate the number of participants with missing data for each variable of interest.	Table 1
14c. Descriptive data – follow-up time	**Cohort study:** Summarise follow-up time—e.g., average and total amount.	Not applicable
15. Outcome data	**Cross-sectional study:** Report numbers of outcome events or summary measures.	Results section, text, Figure 1 and Tables 2, 3 and 4.
16a. Main results	Give unadjusted estimates and, if applicable, confounder-adjusted estimates and their precision (e.g., 95% confidence intervals). Make clear which confounders were adjusted for and why they were included.	Material and Methods, Section 2.4. Statistical analysis. Results section, last paragraph page 4 and Table 5.
16b. Main results – category boundaries	Report category boundaries when continuous variables were categorised.	Material and Methods, Section 2.3, first paragraph; Results (Table 1 and Table 5).
16c. Main results – risk	If relevant, consider translating estimates of relative risk into absolute risk for a meaningful time period.	Not applicable, cross-sectional study
17. Other analyses	Report other analyses done—e.g., analyses of subgroups and interactions, and sensitivity analyses.	Not applicable
**Discussion**		

18. Key results	Summarise key results with reference to study objectives.	Discussion section, paragraphs 1 and 2
19. Limitations	Discuss limitations of the study, taking into account sources of potential bias or imprecision. Discuss both direction and magnitude of any potential bias.	Discussion section, paragraph 7
20. Interpretation	Give a cautious overall interpretation considering objectives, limitations, multiplicity of analyses, results from similar studies, and other relevant evidence.	Discussion section
21. Generalisability	Discuss the generalisability (external validity) of the study results.	Discussion, paragraph 7
**Other information**		
22. Funding	Give the source of funding and the role of the funders for the present study and, if applicable, for the original study on which the present article is based.	Not applicable
